# Early Perihematomal Edema Expansion: Definition, Significance, and Association with Outcomes after Intracerebral Hemorrhage

**DOI:** 10.1155/2021/6249509

**Published:** 2021-09-12

**Authors:** Xin-Ni Lv, Zuo-Qiao Li, Lan Deng, Wen-Song Yang, Yu-Lun Li, Yuan-Jun Huang, Yi-Qing Shen, Xiong-Fei Xie, Xin-Hui Li, Zi-Jie Wang, Zhi-Wei Zhang, Fa-Jin Lv, Jin-Biao Luo, Shu-Jie Sun, Peng Xie, Qi Li

**Affiliations:** ^1^Department of Neurology, The First Affiliated Hospital of Chongqing Medical University, Chongqing 400016, China; ^2^NHC Key Laboratory of Diagnosis and Treatment on Brain Functional Diseases, The First Affiliated Hospital of Chongqing Medical University, Chongqing 400016, China; ^3^Department of Radiology, The First Affiliated Hospital of Chongqing Medical University, Chongqing 400016, China; ^4^Department of Neurosurgery, Guangzhou First People's Hospital, School of Medicine, South China University of Technology, Guangzhou 510180, China; ^5^Department of Neurosurgery, The Xuhui Hospital Affiliated to Fudan University, Shanghai 200031, China

## Abstract

**Objective:**

To investigate the association between early perihematomal edema (PHE) expansion and functional outcome in patients with intracerebral hemorrhage (ICH).

**Methods:**

Patients with ICH who underwent initial computed tomography (CT) scans within 6 hours after the onset of symptoms and follow-up CT scans within 24 ± 12 hours were included. Absolute PHE increase was defined as the absolute increase in PHE volume from baseline to 24 hours. A receiver-operating characteristic (ROC) curve was generated to determine the cutoff value for early PHE expansion, which was operationally defined as an absolute increase in PHE volume of >6 mL. The outcome of interest was 3-month poor outcome defined as modified Rankin scale score of ≥4. A multivariable logistic regression procedure was used to assess the association between early PHE expansion and outcome after ICH.

**Results:**

In 233 patients with ICH, 89 (38.2%) patients had poor outcome at 3-month follow-up. Early PHE expansion was observed in 56 of 233 (24.0%) patients. Patients with early PHE expansion were more likely to have poor functional outcome than those without (43.8% vs. 11.8%, *p* < 0.001). After adjusting for age, admission systolic blood pressure, admission Glasgow Coma Scale score, baseline ICH volume and the presence of intraventricular hemorrhage, and time from onset to CT, early PHE expansion was associated with poor outcome (adjusted odds ratio, 4.25; 95% confidence interval, 1.70–10.60; *p* = 0.002).

**Conclusions:**

The early PHE expansion was not uncommon in patients with ICH and was correlated with poor outcome following ICH.

## 1. Introduction

Spontaneous intracerebral hemorrhage (ICH) is a life-threatening subtype of stroke with high morbidity and disability [1, 2]. Age, admission Glasgow Coma Scale (GCS) score, baseline hematoma size, hemorrhage location, and the presence of intraventricular hemorrhage (IVH) have been well established as predictors of functional outcome after ICH, according to previous studies [3–5]. Perihematomal edema (PHE) is generally considered as a radiological marker for secondary injury after ICH [6]. Several studies have investigated the relationship between PHE volume and outcome after ICH, and the results have been inconsistent [7–10]. Some studies found that PHE volume predicted short-term and long-term outcomes in patients with ICH [7, 9]. Recent studies reported no significant association between PHE and functional outcome [8, 10]. Few studies have focused on the dynamic change in PHE. We aimed to investigate whether early PHE expansion within the first 36 hours could be associated with poor outcome in patients with ICH.

## 2. Subjects and Methods

### 2.1. Study Design

Our study included ICH patients admitted between July 2011 and July 2017 in the First Affiliated Hospital of Chongqing Medical University from our ongoing prospective cohort database. All participants or their legal surrogates were provided written informed consent. This study was conducted in accordance with local ethical frameworks. We recruited ICH patients who had undergone an initial computed tomography (CT) scan within 6 hours after symptoms and a follow-up CT scan within 24 ± 12 hours. Patients with early surgical intervention before follow-up CT scans and those with secondary ICH were excluded. Demographic and clinical characteristics including age, sex, premorbid medical history of hypertension, diabetes mellitus, heart disease, renal disease, smoking status, and premorbid modified Rankin scale (mRS) score were collected for each patient. Detailed data such as the admission blood pressure, time between onset of symptoms and baseline CT scan, and the GCS score were prospectively collected.

### 2.2. Imaging Analysis

We saved Digital Imaging and Communications in Medicine (DICOM) format data of both admission and follow-up CT scans which were collected for further review. ICH and PHE volumes on admission and follow-up CT scans were segmented by a semiautomated computer-assisted volumetric analysis as previously described (Version 11.0; AnalyzeDirect, Overland Park, KS, USA) [11]. In brief, the PHE volume was measured slice by slice on both baseline and follow-up CT scans. All images were evaluated independently by two experienced neurologists in a blinded manner. Hematoma expansion was defined as an increase in hematoma volume of >33% or >6 mL at follow-up CT scan [12, 13]. A semiautomated threshold-based approach (range of 5–33 Hounsfield Units) was applied to identify edematous regions as previously described [14]. The discriminant value of the early PHE expansion was determined by the receiver-operating characteristic (ROC) curve analysis. We chose the optimal cutoff that had the highest sensitivity and specificity for defining early PHE expansion. We calculated the sensitivity and specificity for this dichotomous threshold and used multivariable logistic regression to adjust for potential confounding of important risk factors. Multivariate logistic regression analyses were performed by using variables with *p* < 0.1 in the univariate analysis. Variables known to be related to poor clinical outcome based on several external datasets were also entered into the multivariable model.

### 2.3. Outcome Assessment

The functional outcome was assessed using mRS at 3 months after the onset of symptoms by phone interviews (performed by trained physicians). Poor outcome in this study was defined as 3-month mRS of 4–6.

### 2.4. Statistical Analysis

Data management and analysis were conducted using SPSS software (Version 25.0; IBM Corporation, Armonk, NY, United States). Discrete variables were presented as percentages (%), and the continuous data were expressed as the mean ± standard deviation (SD) or median and interquartile range (IQR), as applicable. The baseline variables, including age, sex, blood pressure, and hematoma volume, were compared with Fisher's exact test, Student's *t* test, or Mann–Whitney *U* test as appropriate. Using univariate logistic regression analysis, clinical and neuroimaging variables were tested for their association with clinical outcome. Furthermore, we utilized ROC curve analyses to determine the early PHE expansion cutoffs and the related sensitivity and specificity in the prediction of 3-month poor outcome. A multivariable logistic regression procedure was used to assess variables. A *p* value < 0.05 was considered statistically significant.

## 3. Results

### 3.1. Baseline Characteristics

**Figure 1:**
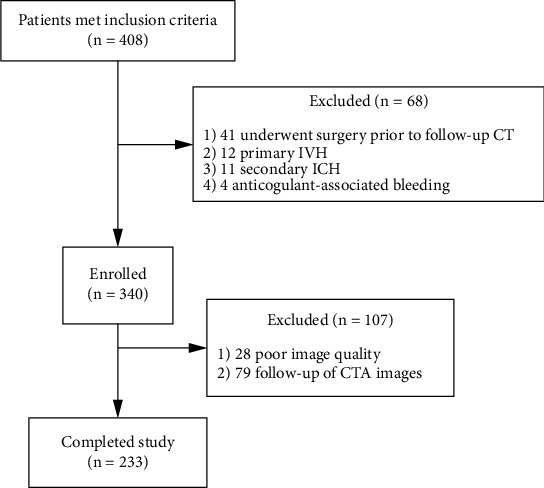
Cohort selection flowchart. IVH: intraventricular hemorrhage; ICH: intracerebral hemorrhage.

A total of 233 patients were included in our final analysis. The flowchart for inclusion is illustrated in Figure 1. The mean age of the participants was 60.2 years (range, 29.0–94.0 years), and 85 patients (36.5%) were women. The median time from symptom onset to the baseline CT scan was 2.0 hours (IQR, 1.0–4.0). The median baseline ICH volume was 13.4 mL (IQR, 8.8–21.1 mL), and the median baseline PHE volume was 6.2 mL (IQR, 3.2–10.5 mL). Intraventricular hemorrhage was observed in 75 of 233 (32.2%) patients on the initial CT scan. In our study population, the baseline hematoma was located in the basal ganglia (*n* = 138, 59.2%), thalamus (*n* = 62, 26.6%), and others (*n* = 33, 14.2%). Patients with poor outcome were more likely to have a larger absolute PHE increase (median 1.5 vs. 3.8 mL, *p* < 0.001). Significant early PHE expansion was present in 56 of 233 (24.0%) patients on follow-up CT scan. The baseline demographic and clinical characteristics are described in Table 1.

### 3.2. Receiver-Operating Characteristic Analysis

Univariate ROC analysis revealed a high diagnostic performance for early PHE expansion in the discrimination of good versus poor outcome after ICH with an optimal cutoff of 6.83 (area under the curve, AUC 0.704, 41.6% sensitivity, 90.3% specificity). To facilitate clinical evaluation, we considered a value of 6 mL as the optimal threshold for early PHE expansion which was determined by ROC curve analyses. Early PHE expansion > 6 mL was observed in 56 (24.0%) patients. The sensitivity and specificity value of PHE expansion > 6 mL for predicting poor outcome at 3 months were 43.8% and 88.2%, respectively.

### 3.3. Predictors of Functional Outcome in ICH Patients

**Figure 2:**
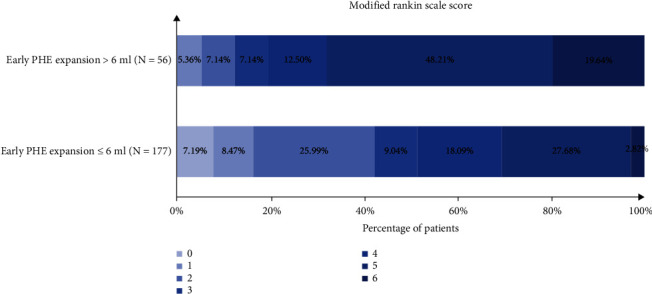
Distribution of modified Rankin scale score in patients with or without early PHE expansion. PHE: perihematomal edema.

A total of 89 (38.2%) patients had poor outcome (mRS 4–6) at 3-month follow-up (Table 2). After adjusting for age, admission systolic blood pressure, admission GCS score, baseline hematoma volume and the presence of intraventricular hemorrhage, and time from onset to CT, early PHE expansion was associated with poor outcome (adjusted odds ratio (OR), 4.25; 95% confidence interval (CI), 1.70–10.60; *p* = 0.002) (Table 2). The distribution of 3-month mRS in patients with and without early PHE expansion is shown in Figure 2.

### 3.4. Factors Associated with PHE Expansion

Univariate and multivariate logistic regression analyses were performed to assess the association between various parameters and early PHE expansion (Table 3). In the univariate analysis, the admission systolic blood pressure, admission GCS score, baseline hematoma volume, and time from onset to CT were associated with PHE expansion > 6 mL (all *p* values < 0.1). In multivariable logistic regression analysis, systolic blood pressure (OR, 1.02; 95% CI, 1.00–1.03; *p* = 0.022), baseline hematoma volume (OR, 1.11; 95% CI, 1.07–1.15; *p* < 0.001), and the time from onset to CT (OR, 0.59; 95% CI, 0.44–0.78; *p* < 0.001) remained independent predictors of early PHE expansion.

## 4. Discussion

We demonstrated that an absolute PHE increase correlated with the 3-month clinical outcome in ICH patients. Moreover, our selected dichotomous threshold, early PHE expansion > 6 mL, was strongly associated with poor clinical outcome after adjusting for potential confounding factors.

Previous reports investigated the association between PHE increase and outcome when analyzing PHE evolution up to 24 or 72 hours in ICH patients [15–17]. A pooled analysis of the Intensive Blood Pressure Reduction in Acute Cerebral Haemorrhage Trial (INTERACT) 1 and 2 trials showed that early absolute PHE increase up to 24 hours after onset was associated with increased 90-day odds of death or dependency [15]. A recent analysis of ICH patients from the Virtual International Stroke Trials Archive-ICH (VISTA-ICH) study (*n* = 596) demonstrated that 72-hour absolute PHE volume growth was independently associated with poor outcome in the basal ganglia ICH patients or those with hematoma volumes < 30 mL [16]. A retrospective single-center study found that a 24-hour absolute PHE increase was independently associated with mortality and functional outcome in ICH patients [17]. Several studies have investigated the association between relative PHE (defined as PHE volume/hematoma volume) and functional outcome [7, 8, 10, 18]. An early study concluded an inverse correlation between a larger relative edema volume and worse 12-week functional outcome [10]. However, subsequent studies have failed to identify an association between relative PHE and outcomes [7, 8, 18]. Consistent with previous studies, we also demonstrated that an absolute PHE increase was independently associated with poor outcome. However, there are no well-established criteria for early PHE expansion in patients with ICH. In our study, we proposed a cutoff value for the diagnosis of early PHE expansion on CT based on ROC curves. Early PHE expansion was operationally defined as an increase in its volume by >6 mL. We found that early PHE expansion occurred in 56 of 233 (24.0%) ICH patients. Since early PHE expansion is related to poor outcome after ICH, identifying patients with PHE may be useful for designing treatment trials to mitigate early PHE expansion.

Previous studies suggested that hematoma expansion (HE) mostly occurred within 3 hours after the onset of symptoms and was rare after 24 hours [3, 4]. Unlike early hematoma growth, 2-stage edema PHE expansion has been reported in recent observational studies [19]. The early phase of PHE expansion usually develops as early as 3 hours after the onset of symptoms and increases over time by approximately 100% during the first 24 hours [15–17]. The PHE expansion rate was defined as the PHE volume increase divided by the time interval between the baseline and the follow-up scans [17]. Several studies have investigated the association between PHE expansion rate and functional outcome in ICH patients [17, 20]. In a study of 139 patients, Urday et al. reported that a higher 24-hour edema expansion rate after ICH was associated with worse outcome [17]. A recent retrospective analysis showed that the edema expansion rate over the first 24 hours correlated with death or disability for both lobar and deep spontaneous ICH. However, the 72-hour PHE expansion rate was associated with worse functional outcome only in deep ICH patients [20]. In addition, late-phase PHE growth occurs between 2 and 4 weeks after the ICH event. Animal and in vitro studies have suggested that the development of brain edema is activated by an increase in the production of proinflammatory and anti-inflammatory mediators within hours after stroke [21, 22]. This correlation between the time from symptom onset to CT and PHE evolution in the acute process was confirmed in our study. Therefore, we have used an early timeframe for PHE assessment to capture PHE expansion. Recent analysis of the Antihypertensive Treatment of Acute Cerebral Hemorrhage-2 (ATACH-2) study suggested that ultraearly (<2 hours after onset) blood pressure reduction was associated with reduced HE and improved outcome in ICH patients [23]. Therapy focusing on reducing HE or PHE expansion may be effective within the early therapeutic time-window. In our study, we observed that 24.0% of patients experienced PHE expansion within 6 hours after the onset of symptoms. We also demonstrated that early PHE expansion was associated with poor outcome in ICH patients. Since early PHE expansion may be a promising target for intervention, early identification of PHE and initiation of antiedema expansion treatment may be useful in the management of ICH patients.

In patients with ICH, previous studies defined hematoma growth as a 33% increase in hematoma volume or >6 mL at follow-up CT scan in previous studies [12, 13]. Recently, Yogendrakumar et al. proposed revised criteria for defining HE in the ICH populations [24]. However, there are no well-established criteria for defining PHE expansion. In previous studies, an absolute PHE volume increase was associated with poor outcome in patients with ICH. In the present study, we proposed a quantitative criterion for PHE expansion in the early phase. Therefore, strategies targeting this early phase of PHE expansion are likely to alter the edema growth trajectory with reduced peak edema and may result in improved outcome.

In our study, we also observed that baseline hematoma volume was independently associated with early PHE expansion. From a pathophysiological standpoint, the components of the hematoma may influence PHE evolution [25–28]. Studies have also indicated that the surrogate markers of iron load such as the serum ferritin level and hematocrit are considered to be the relevant factors for edema formation in ICH [25–27]. After erythrocyte lysis, the iron concentration in the brain could reach as high as 10 mmol/L, causing the thrombin-induced brain edema [25]. A recent study showed that a higher admission hematocrit was associated with a greater delay in peak PHE [28]. Theoretically, a larger hematoma size produces a more absolute PHE increase [29]. These findings may indicate a dose effect of blood components and breakdown products on PHE formation.

Our study has several limitations. First, only patients from one single center were included. Second, our study only investigated the dynamic PHE change during the acute phase of ICH. However, PHE eveolution is a biphasic event, and the late-phase PHE changes are still unknown. Future studies with larger sample sizes and follow-up scans at different time points are needed to further clarify the natural history of PHE evolution.

## 5. Conclusions

In summary, we have demonstrated that an absolute PHE increase independently predicted poor outcome in patients with ICH. Early PHE expansion > 6 mL is an optimal threshold associated with poor outcome in patients with spontaneous ICH within 6 hours of onset. Early PHE expansion may aid in the selection of new therapeutic strategies and patient groups most likely to benefit from intervention. Future studies are needed to determine the effect of anti-PHE expansion treatment in limiting PHE growth.

## Figures and Tables

**Table 1:** Comparison of baseline demographic, clinical, and radiological characteristics between patients with and without favorable outcome.

Variables	Patients, no. (%)		
	Favorable outcome (*n* = 144, 61.8%)	Poor outcome (*n* = 89, 38.2%)	*p* value
*Demographic*			
Mean age, y (SD)	**58.6 (11.5)**	**62.7 (12.4)**	**0.011**
Sex, male, *n* (%)	87 (60.4)	61 (68.5)	0.211
*Medical history*			
Alcohol consumption, *n* (%)	57 (39.6)	38 (42.7)	0.638
Smoking, *n* (%)	61 (42.4)	44 (49.4)	0.291
Hypertension, *n* (%)	103 (71.5)	71 (79.8)	0.160
Diabetes mellitus, *n* (%)	14 (9.7)	12 (13.5)	0.376
*Clinical features*			
Systolic blood pressure, mmHg (SD)	167.5 (26.0)	176.6 (35.0)	0.035
Diastolic blood pressure, mmHg (SD)	97.3 (14.5)	101.2 (21.6)	0.128
Admission GCS score, median (IQR)	**14 (13-15)**	**10 (7-14)**	**<0.001**
Baseline ICH volume, mL (IQR)	**11.7 (7.1-16.4)**	**18.7 (11.1-32.0)**	**<0.001**
Time from onset to CT, h(IQR)	**2.0 (1.0-4.0)**	**2.0 (1.0-3.0)**	**0.046**
Admission PHE volume, mL (IQR)	**5.3 (2.6-8.3)**	**7.5 (4.7-14.7)**	**<0.001**
Follow-up PHE volume, mL (IQR)	**7.8 (4.0-11.1)**	**13.7 (7.0-25.1)**	**<0.001**
Absolute PHE increase, mL (IQR)	**1.5 (0.1-3.5)**	**3.8 (1.2-11.9)**	**<0.001**
Early PHE expansion, *n* (%)	**17 (11.8)**	**39 (43.8)**	**<0.001**
SAH at baseline CT, *n* (%)	11 (7.6)	9 (10.1)	0.513
IVH at baseline CT, *n* (%)	**28 (19.4)**	**47 (52.8)**	**<0.001**
Hematoma growth, *n* (%)	**27 (18.8)**	**45 (50.6)**	**<0.001**
ICH location			
Basal ganglia hemorrhage, *n* (%)	**95 (66.0)**	**43 (48.3)**	**0.008**
Thalamic hemorrhage, *n* (%)	**29 (20.1)**	**33 (37.1)**	**0.004**
Other, *n* (%)	20 (13.9)	13 (14.6)	0.879
*Outcome*			
In-hospital mortality, *n* (%)	**0 (0)**	**16 (18.0)**	**<0.001**
30-day mortality, *n* (%)	**0 (0)**	**33 (37.1)**	**<0.001**
90-day mRS, median (IQR)	**1 (0-2)**	**6 (4.5-6)**	**<0.001**

ICH: intracerebral hemorrhage; CT: computed tomography; IVH: intraventricular hemorrhage; SAH: subarachnoid hemorrhage; GCS: Glasgow Coma Scale; mRS: modified Rankin scale; PHE: perihematomal edema; IQR: interquartile range; and SD: standard deviation.

**Table 2:** Univariate and multivariate analysis of predictors for poor outcome (mRS 4-6) at 3 months.

Variable	Odds ratio	95% confidence interval	*p* value
Univariate analysis			
Age, year^a^	1.03	1.01-1.05	**0.012**
Sex, male	1.43	0.82-2.50	0.212
Alcohol consumption	1.14	0.67-1.95	0.639
Smoking	1.33	0.78-2.26	0.292
Hypertension	1.57	0.84-2.95	0.161
Diabetes mellitus	1.45	0.64-3.29	0.378
Systolic blood pressure, mmHg^a^	1.01	1.00-1.02	**0.025**
Diastolic blood pressure, mmHg^a^	1.01	1.00-1.03	**0.097**
Admission GCS score^a^	0.69	0.62-0.77	**<0.001**
Baseline ICH volume, mL^a^	1.07	1.04-1.10	**<0.001**
IVH at baseline CT	4.64	2.58-8.33	**<0.001**
Time from onset to CT, hour^a^	0.84	0.71-1.00	**0.046**
Admission PHE volume, mL^a^	1.12	1.06-1.17	**<0.001**
Absolute PHE increase, mL^a^	1.16	1.09-1.23	**<0.001**
Early PHE expansion	5.83	3.02-11.24	**<0.001**
Multivariate analysis			
Age, year^a^	1.03	1.00-1.06	0.073
Systolic blood pressure, mmHg^a^	1.01	0.99-1.02	0.289
Admission GCS score^a^	0.76	0.68-0.86	**<0.001**
Baseline ICH volume, mL^a^	1.06	1.02-1.11	**0.003**
IVH at baseline CT	5.41	2.48-11.81	**<0.001**
Time from onset to CT, hour^a^	0.98	0.78-1.24	0.852
Early PHE expansion	4.25	1.70-10.60	**0.002**

ICH: intracerebral hemorrhage; CT: computed tomography; IVH: intraventricular hemorrhage; GCS: Glasgow Coma Scale; mRS: modified Rankin scale; PHE: perihematomal edema; OR: odds ratio; CI: confidence interval. ^a^Per unit change in regressor.

**Table 3:** Univariate and multivariate analysis of predictors for early PHE expansion.

Variable	Odds ratio	95% confidence interval	*p* value
Univariate analysis			
Age, year^a^	1.00	0.97-1.02	0.839
Sex, male	1.60	0.83-3.07	0.160
Alcohol consumption	1.49	0.82-2.73	0.195
Smoking	1.73	0.94-3.16	0.077
Hypertension	1.02	0.51-1.96	0.949
Diabetes mellitus	1.19	0.47-2.99	0.715
Systolic blood pressure, mmHg^a^	1.02	1.01-1.03	**0.005**
Admission GCS score^a^	0.86	0.78-0.93	**<0.001**
Baseline ICH volume, mL^a^	1.10	1.06-1.13	**<0.001**
IVH at baseline CT	0.89	0.47-1.71	0.736
Time from onset to CT, hour^a^	0.66	0.52-0.83	**<0.001**
Multivariate analysis			
Systolic blood pressure, mmHg^a^	1.02	1.00-1.03	**0.022**
Admission GCS score^a^	0.95	0.85-1.06	0.388
Baseline ICH volume, mL^a^	1.11	1.07-1.15	**<0.001**
Time from onset to CT, hour^a^	0.59	0.44-0.78	**<0.001**

ICH: intracerebral hemorrhage; CT: computed tomography; IVH: intraventricular hemorrhage; GCS: Glasgow Coma Scale; PHE: perihematomal edema; OR: odds ratio; CI: confidence interval. ^a^Per unit change in regressor.

## Data Availability

The datasets presented in this article are not readily available because the datasets generated for this study are available on request to the corresponding author.
